# Long-term care use and socio-economic status in Belgium: a survival analysis using health care insurance data

**DOI:** 10.1186/0778-7367-71-1

**Published:** 2013-01-03

**Authors:** Karel Van den Bosch, Joanna Geerts, Peter Willemé

**Affiliations:** 1 Belgian Federal Planning Bureau, Kunstlaan 47-49, Brussel, 1000, Belgium

**Keywords:** Long-term care, Socio-economic status, Morbidity, Mortality, Preferential status

## Abstract

**Background:**

The small but growing literature on socio-economic inequality in morbidity among older persons suggests that social inequalities in health persist into old age. A largely separate body of literature looks at the predictors of long-term care use, in particular of institutional care. Various measures of socio-economic status are often included as control variables in these studies. Review articles generally conclude that the evidence for such variables being a predictor for institutionalization is “inconclusive”. In this paper we look at the association among older persons in Belgium between one particular measure of socio-economic status – preferential status in public health care insurance – and first use of home long-term care and residential care. Preferential status entitles persons to higher reimbursement rates for health care from the public health care insurance system and is conditional on low income. We also study whether preferential status is related to the onset of five important chronic conditions and the time of death.

**Methods:**

We use survival analysis; the source of the data is a large administrative panel of a sample representative for all older persons in Belgium (1,268,740 quarterly observations for 69,562 individuals).

**Results:**

We find a strong association between preferential status and the likelihood of home care use, but for residential care it is small for men and non-existent for women. We also find that preferential status is significantly related to the chance of getting two out five chronic conditions – COPD and diabetes, but not dementia, hip fracture and Parkinson’s disease – and to the probability of dying (not for women). For home care use and death, the association with preferential status declines with increasing age from age 65 onwards, such that it is near zero for those aged around 90 and older.

**Conclusion:**

We find clear associations between an indicator of low income and home care use, some chronic conditions and death. The associations are stronger among men than among women. We also find that the association declines with age for home care use and death, which might be explained by selective survival.

## Background

A voluminous literature has established that persons with low socio-economic status have worse health than those with better socio-economic status. This has also been found for Belgium [1]. Most of this research has focused on working-age adults [2]. A small but growing literature on socio-economic inequality in morbidity among older persons suggests that social inequalities in health persist into old age [2,3]. Avendano et al. [3] for example, find that “lower educational level is associated with higher incident events of poor health, chronic diseases and disability, but it is less consistently associated with new events of long-standing illness.” A recent study for Belgium [4] has found that frailty among Belgian elderly persons is associated with their socio-economic status (confirming other research [5]) and is strongly associated with their health- and home-care utilization. There is discussion about the most appropriate measure of socio-economic status for older persons [2,6]. With regard to health status, most studies use fairly general indicators of overall health (e.g. self-assessed health, disability), and do not focus on specific conditions.

A largely separate body of literature looks at the predictors of long-term care use, in particular of institutional care. Predictors often include socio-economic variables, such as education, income, wealth and home-ownership. For an overview of the effects of these predictors, we rely mainly on recent review articles [7,8], supplemented by some studies published subsequently. Gaugler et al. [7] conclude that the evidence for education being a predictor for nursing home placement is ‘inconclusive’. When a significant impact of education is found, the direction of the effect varies [9-11]. In all cases, the effects are not very strong. A few studies look at the effect of a person’s or family’s wealth on the probability of institutionalization, without conclusive results [9,12,13]. By contrast, consistent results are found for home-ownership: the evidence is strong that homeowners are much less likely than others to enter an institution, though it is not clear how this finding should be interpreted [7-9,11,14,15].

The evidence that income predicts nursing home placement is ‘inconclusive’ [8]. The meta-analysis by Gaugler et al. [7] indicates that low income is an important (positive) predictor of institutionalisation, whereas results of other studies are mixed [9,10,16]. Generally, the relation between income and long-term care use seems to be interpreted in terms of income and price effects, though this issue is not given much attention. The income effect refers to the possibility that persons with a higher income might find it easier to pay for long-term care, and might therefore, *ceteris paribus*, be more inclined to enter residential care. On the other hand, a higher income might also facilitate access to home care services and might therefore assist in delaying residential care entry. Price effects come into play when out-of-pocket payments for long-term care are somehow dependent on income. In Finland, for example, user charges for institutional care are related to disposable income, making it in absolute terms much more expensive for individuals with a high income, and providing an economic incentive for those persons to avoid long-term institutionalization [10]. Where costs of long-term care are covered by public programs for persons with low incomes (e.g. Medicaid in the United States), the latter might be more inclined to enter an institution than those who have to pay all or a larger part of these costs out of their own pocket [17]. Such institutional differences between countries are no doubt one reason for the variable results across studies. The association between income and long-term care use is less often interpreted in terms of the socio-economic gradient in health. One consequence or indicator of low socio-economic status is low income, and if such persons experience worse health, they might have a greater need for long-term care, leading to an increased likelihood of using such care. Presumably it is assumed that the statistical association resulting from this mechanism is controlled for by the inclusion of one or more measures of health and/or disability. Still, there might be unobserved heterogeneity in health between older persons with varying levels of income.

In this paper we look at the association among older persons in Belgium between one particular measure of socio-economic status – preferential status in health care insurance – and first observed use of home long-term care and residential care. We also examine its relationship with the onset of five important chronic conditions (COPD, dementia, diabetes, hip fracture and Parkinson’s disease) and with death. We use a large administrative panel of a sample representative for all older persons in Belgium. The large sample size and the fact that we have quarterly observations for the period 2004–2009 make it possible to use survival analysis techniques, which take into account the timing of the events at issue here.

## Methods

The source of the data is the ‘Echantillon Permanent(e) Steekproef’ (Permanent Sample, EPS), a large administrative panel of a sample of all persons within the Belgian public health insurance [18]. The latter covers virtually all persons resident in Belgium. The exceptions are mostly recent immigrants, of which there are few among older men and women. We use data for persons aged 65 and over only, for whom the sampling fraction is 5%. The EPS contains all information (suitably anonymized) that is available to the public health insurance agencies (the sickness funds and the National Institute for Health and Disability Insurance), which includes use of acute and long-term medical care and medicines, as well as some variables related to the socio-economic situation of insured persons. Health status and health problems as such are not registered, though. However, we could identify persons suffering from one or more of five chronic conditions (COPD, dementia, diabetes, hip fracture and Parkinson’s disease) by looking at the use of medicines or kinds of medical care that are specific to those conditions. These conditions are important predictors of disability. Unfortunately, the imputation of those conditions is not complete, since it is known that many persons suffering from diabetes are undiagnosed as such, while many persons with severe symptoms of dementia do not receive medication. We use data for the years 2004–09.

None of the measures of socio-economic status (education, occupation, income, wealth, home tenure) commonly used in the literature are available in the EPS. We use “preferential status” in the public health insurance as a proxy for income. Persons with preferential status enjoy higher reimbursements (lower co-payments) for many health care transactions, as well as some other advantages. Low income is a requirement for obtaining this preferential status. ^a^ For persons living on some types of means-tested income benefits the low income requirement is waived, as it is in fact implicit in the means test. However, a serious complication is that the low income requirement is also waived for older persons receiving a benefit because of disability. While this particular benefit is also means-tested, the means test is less strict than the one applied for non-disabled older persons. For this reason, and to avoid endogeneity (or reverse causation), only persons for whom there was no administrative indication of disability or handicap at the first quarter when they were observed (i.e. in the 1st quarter of 2004, or the first quarter of the year when they turned 65) were included in the analysis. More exactly, initially their status in social insurance is not “disabled”, the person is not officially recognized as “disabled”, and she or he had no certificate of chronic illness or benefit for handicapped persons. While this does not mean that those persons had no health problem at all, it excludes all or nearly all persons who enjoyed preferential status because of disability or chronic conditions, without necessarily having low income. Also excluded were those suffering from one of the chronic conditions at the initial period, as well as persons using any form of long-term care at that time. Finally, before 2008 many formerly self-employed persons had no public health insurance for « minor risks », which include home care and the lump-sum payments for residential care in homes for the elderly; such persons were only covered for long-term care in nursing homes. Since their pattern of long-term care use is likely to be quite different from that of the rest of the population, we excluded those persons.

We use survival analysis (also known as event-history analysis) to estimate the association of preferential status with morbidity, death and long-term care use, as this method makes the most optimal use of the panel data at hand with time-dependent covariates and censoring of many cases [19]. Given the large sample and quarterly observations, there are of course a very large number of ties (where several individuals experience the event of interest at the same moment in time), making application of Cox regression models problematic. For this reason we present results of discrete survival analyses, using logistic regression [20]. For each situation or condition of interest (home care use, residential care use, COPD, dementia, diabetes, hip fracture, Parkinson’s disease and death), a separate survival analysis was performed. As a sensitivity test, the final models were also estimated with a Cox proportional hazard model, using the Efron approximation for tied data, and the results were very similar (see Additional file 1).

Age, province, living with a partner or not and dummy variables for each year and each quarter were included as controls. In order to retain maximum flexibility of functional form, and given the large sample size, age was entered with a dummy for each age in years. Province (some larger provinces were split up) was included because the supply of long-term care varies across provinces, and there are regional differences in morbidity and mortality among older persons in Belgium [21]. Living with a partner strongly reduces the chances to enter residential care [7,13], and is also associated with better health [22]. All analyses were performed separately for women and men, as patterns of chronic conditions and long-term care use may well differ by sex.

Table 1 shows that the total number of individuals in the sample selected for analysis is 69,562, while 36,665 persons are excluded. Given an average of 18.2 observed quarters per individual, this produces a total of 1,268,740 observations of person-quarters. Nearly all individuals who are initially older than 65 enter the sample in 2004 (the exceptions are immigrants and persons coming back into public health insurance), while a substantial number of persons are first observed in later years, when they turn 65. The maximum number of quarters for which persons can be observed is 22 (for technical reasons observations in the first quarter of 2004 and the last quarter of 2009 could not be used). Among the youngest age group, many persons enter the sample later than 2004, while among the older age groups, the observation period is often cut short by death. For all analyses, except for death, the total number of observations is in fact lower than the numbers mentioned above, since survival analysis does not use observations (quarters) after the first occurrence of the condition or situation at issue. The exact number of observations used in each analysis can be found in Additional file 2.

**Table 1:** Characteristics of persons aged 65 or more within the permanent sample of persons covered by the Belgian public health insurance (2004–09), sample selected for analysis and excluded cases

		**Men**			**Women**		**All**
**Age initially**	**65-74**	**75-84**	**85+**	**65-74**	**75-84**	**85+**	
**Analysis sample (a)**							
Preferential status initially	17.2%	24.5%	35.1%	24.2%	36.0%	46.4%	24.7%
COPD*	7.8%	11.3%	8.0%	5.7%	7.0%	7.2%	7.3%
Dementia*	2.9%	8.6%	10.5%	4.0%	10.8%	14.6%	5.7%
Diabetes*	5.9%	5.6%	2.8%	5.1%	5.0%	2.7%	5.3%
Hip fracture*	2.1%	4.4%	6.6%	3.5%	8.7%	14.3%	4.3%
Parkinson’s disease*	1.2%	3.2%	2.1%	1.2%	2.6%	2.6%	1.7%
Dead*	7.3%	25.1%	59.9%	3.6%	15.7%	43.0%	11.1%
Home care*	3.3%	15.9%	25.3%	5.9%	22.9%	35.8%	10.0%
Residential care*	1.0%	7.8%	23.9%	1.3%	13.3%	34.1%	5.1%
Partner**	71.3%	62.6%	37.9%	57.2%	26.5%	6.5%	55.6%
Partner loss*	7.6%	11.0%	14.0%	8.1%	13.0%	6.6%	9.1%
Partner gained*	2.6%	1.6%	1.8%	1.1%	0.5%	0.6%	1.5%
Single**	18.5%	24.7%	46.2%	33.5%	60.0%	86.3%	33.7%
Start year is 2004	63.8%	99.6%	99.7%	66.0%	99.5%	99.1%	75.9%
Number of observed quarters	17.2	19.5	15.6	17.9	20.5	17.6	18.2
Number of individuals	21,894	7,840	1,039	25,607	11,301	1,881	69,562
**Excluded cases**							
Preferential status initially	33.7%	41.6%	58.6%	44.5%	55.7%	66.8%	47.5%
COPD*	39.9%	42.7%	30.2%	28.5%	25.2%	16.4%	30.8%
Dementia*	14.0%	21.1%	21.9%	20.1%	28.7%	29.7%	22.1%
Diabetes*	43.9%	32.5%	19.1%	41.5%	32.8%	16.4%	35.0%
Hip fracture*	4.3%	7.4%	9.2%	7.2%	13.8%	13.3%	8.9%
Parkinson’s disease*	6.0%	11.2%	8.2%	6.7%	10.4%	8.1%	8.2%
Dead*	23.0%	55.0%	81.0%	14.8%	39.8%	72.2%	37.1%
Home care*	13.8%	33.9%	39.2%	22.2%	42.7%	34.3%	28.7%
Residential care*	6.9%	23.2%	49.5%	9.6%	39.2%	66.3%	25.9%
Partner**	65.0%	56.4%	29.4%	48.6%	19.2%	3.5%	40.4%
Partner loss*	6.7%	9.5%	8.4%	8.8%	9.4%	2.9%	7.8%
Partner gained*	2.6%	1.6%	2.1%	1.3%	0.7%	0.4%	1.4%
Single**	25.7%	32.4%	60.1%	41.3%	70.7%	93.2%	50.3%
Start year is 2004	67.6%	99.5%	99.8%	69.9%	99.7%	99.7%	84.8%
Number of observed quarters	16.0	15.7	11.3	17.2	17.7	13.0	16.1
Number of individuals	8,328	4,891	1,282	9,255	8,406	4,503	36,665

(a) at the first observerd quarter: no administrative indication of disability or handicap, not suffering from any of the chronic conditions, not using any form of long-term care.

* Ever during observation period. ** During whole observation period.

Table 1 also shows that older persons and women are more likely to enjoy preferential status. COPD, dementia and diabetes are fairly common chronic conditions. Hip fracture occurs rather frequently among older women, while Parkinson’s disease is less prevalent. The probability of ever having experienced dementia or hip fracture increases strongly with age, which is not true for the other conditions. Unsurprisingly, older people are also more likely to use home care and especially residential care. The selection criteria imply that excluded individuals are much more likely than the selected sample to have preferential status, to suffer from one or more chronic diseases, and to use long-term care. The differences are often more marked in the groups 65–74 and 75–84. The selection procedure has the implication that much of the effect of socio-economic status on health, in so far as it materializes before persons can enter the sample, is bracketed out of the analysis. In this sense, the selection procedure loads the dice against finding an association between preferential status, chronic conditions and long-term care use in this study.

## Results

We first present results for the chronic conditions and death (Table 2), followed by those for home care and residential care (Tables 3 and 4). In each table, to save space, only the coefficients for preferential status are shown; the full results for all predictors can be found in Additional files 2, 3 and 4.

**Table 2:** Association between preferential status and the first occurrence of five chronic conditions and death within the permanent sample of persons covered by the Belgian public health insurance (2004–09)

**Dep. variable**	**Model**	**Men**				**Women**	
		**Est.**	**St.error**	**Sign.**	**Est.**	**St.error**	**Sign.**
COPD		0.317	0.046	0.000	0.250	0.081	0.002
Dementia		0.074	0.064	0.247	0.125	0.099	0.205
Diabetes		0.237	0.059	0.000	0.381	0.091	0.000
Hip fracture		−0.023	0.088	0.792	0.074	0.108	0.496
Parkinson’s disease		0.079	0.108	0.466	0.273	0.191	0.152
Death		0.152	0.036	0.000	0.095	0.036	0.009
Death	interaction with age						
	dummy preferential status:	0.378	0.082	0.000	0.052	0.096	0.589
	interaction variable*:	−0.015	0.005	0.002	0.002	0.005	0.629
Death	with chronic conditions and						
	interaction with age						
	dummy preferential status:	0.381	0.082	0.000	0.055	0.096	0.563
	interaction variable*:	−0.016	0.005	0.001	0.002	0.005	0.636

Coefficients estimated by discrete survival analysis using logistic regression.

Also included in all models: age, partner (time-dependent), province, year, quarter.

* interaction variable is specified as preferential_status * (age – 65).

**Table 3:** Association between preferential status and the first occurrence of home care use within the permanent sample of persons covered by the Belgian public health insurance (2004–09)

**Model**	**Variable**	**Men**			**Women**		
		**Est.**	**St.error**	**Sign.**	**Est.**	**St.error**	**Sign.**
Basic model	Pref. Status	0.266	0.048	0.000	0.089	0.032	0.005
Interaction with age	Pref. Status	0.631	0.126	0.000	0.265	0.081	0.001
	Interaction var*	−0.023	0.008	0.002	−0.011	0.005	0.018
With chronic conditions & interaction with age	Pref. Status	0.575	0.127	0.000	0.255	0.081	0.002
	Interaction var*	−0.020	0.008	0.009	−0.011	0.005	0.029

Coefficients estimated by discrete survival analysis using logistic regression.

Also included in all models: age, partner (time-dependent), province, year, quarter.

* interaction variable is specified as preferential_status * (age – 65).

**Table 4:** Association between preferential status and the first occurrence of use of residential care within the permanent sample of persons covered by the Belgian public health insurance (2004–09)

**Model**	**Variable**	**Men**			**Women**		
		**Est.**	**St.error**	**Sign.**	**Est.**	**St.error**	**Sign.**
Basic model	Pref. Status	0.119	0.070	0.090	0.015	0.043	0.724
Interaction with age	Pref. Status	0.499	0.208	0.017	0.031	0.146	0.831
	Interaction var	−0.021	0.011	0.057	−0.001	0.007	0.909
With chronic conditions	Pref. Status	0.485	0.212	0.022	0.037	0.147	0.799
	Interaction var	−0.022	0.011	0.048	−0.003	0.007	0.725

Coefficients estimated by discrete survival analysis using logistic regression.

Also included in all models: age, partner (time-dependent), province, year, quarter.

* interaction variable is specified as preferential_status * (age – 65).

We find significant and substantial effects of preferential status on the probability of getting COPD and diabetes, both for men and women. No significant effects are observed for dementia, hip fracture and Parkinson’s disease. We also find a significant effect of preferential status on mortality, which is substantially larger for men than for women. Interestingly, the effect of preferential status weakens strongly as persons become older, as shown by the model labelled ‘interaction with age’. The interaction variable is specified in such a way that the coefficient for the dummy variable for preferential status is an estimate of the effect of this status at age 65. (We tried other specifications than the linear one used here, but none produced a significant improvement in model fit). The size of the coefficient for the interaction variable indicates that the effect of preferential status on the probability of death becomes nil when men are aged around 90. For women the interaction effect is not at all significant, however. (We also ran models with a similar age-interaction term for the chronic conditions, but this turned out not to be significant in any case). In the final model with death as the dependent variable, dummies for five chronic conditions are included in the model as time-dependent variables. Surprisingly, this does not at all reduce the estimated effect of preferential status and its interaction with age. This is partly due to the fact that preferential status has no significant association with those chronic conditions which are the strongest predictors of death (hip fracture and dementia). Moreover, persons suffering from (or, rather, being treated for) diabetes are actually *less* likely to die than those without (treatment for) diabetes.

Preferential status also has a strong effect on home care use for both sexes, although the effect is again much larger for men than for women. As was true for death, the model including an interaction term with age (the specification is the same as in the model for death) shows that the effect declines with age, and becomes near zero at age 90, both for men and for women. When dummies for five chronic conditions are included in the model, the estimates of the effect of preferential status and its interaction with age become smaller, though the difference is small for men and negligable for women. This indicates that those five chronic conditions play only a limited role in mediating the association of preferential status with home care use. The main reason for this is that the conditions that are related to preferential status (COPD and diabetes), have only a moderate effect on the use of home care, in contrast to dementia, hip fracture and Parkinson’s disease. Among men, the effect of preferential status on use of residential care is much smaller than it is for home care, and the effect is non-existent for women. Interestingly, the effect is significant only when the interaction term with age is also included. As was true for home care, and for similar reasons, the inclusion of dummies for chronic conditions in the model does not make much difference, though the coefficient for the interaction term becomes significant for men.

## Discussion

We have found an association between preferential status and the likelihood of getting two out five chronic conditions – COPD and diabetes, but not dementia, hip fracture and Parkinson’s disease – and also with the probability of dying. We also found that preferential status is strongly related with home care use. For residential care the relationship is weak for men and non-existent for women. For death and home care use, the association with preferential status declines with age, such that (within the population studied) it is strongest for those aged 65, and near zero for those aged around 90 and older.

As explained in the methods section, we interpret (initial) preferential status, which is conditional on low income, as a measure of socio-economic status. The observed effects of preferential status on COPD, diabetes and death can then be interpreted as instances or consequences of socio-economic differences in morbidity and mortality. A discussion of the possible mechanisms which could be responsible for these differences is beyond the scope of this paper (see for example [3] for a review). For COPD and diabetes, it is plausible that smoking, unhealthy food and other life-style factors could be involved. The fact that preferential status is a dichotomy is an important limitation of our study, as it makes it impossible to find a gradient in its association with chronic conditions and long-term care use. Another limitation is that the presence of chronic conditions is not observed directly, but imputed on the basis of medicines or medical care use. Some medicines or treatments might be cheaper for patients with preferential status than for others.

An interesting finding is that the effect of preferential status on mortality is not mediated by the five chronic conditions that could be identified in the data, even though most of those conditions are shown to be important predictors of death. This suggests that other health problems play a role here, with heart problems being a prime candidate. Unfortunately, the data that would allow us to check this hypothesis are lacking. In a similar vein, we interpret the observed effect of preferential status on home care use (and for men on use of residential care) as a consequence of the worse health of persons with low incomes, given age, sex, living situation and province of residence. Yet, this supposed worse health is captured to only a limited extent by the five chronic conditions mentioned.

We have found that the effect of preferential status declines with increasing age, both for death and for home care use. One must be careful with the interpretation of such interaction effects in logistic models, since they can be an artefact of the functional form chosen [23]. If a linear specification (without interaction terms) would in fact be correct, then interaction terms might well be significant if the model is estimated using a logistic equation. However, other analyses not shown here indicate that the effect of preferential status on death and home care is indeed fairly substantial at ages 65–75, and not only not significant, but also near zero at ages over 85. This is true when this effect is measured in terms of odds-ratio’s (which we use implicitly when applying logistic regression) and also when we look at simple differences between rates. One possible interpretation of this finding is in terms of survivalship bias, or selective mortality. Suppose that the population is in fact composed of two groups, one at high risk of death (say, because of heart problems), and another one at low risk, but that membership of these groups is not observed. Among persons with preferential status, the high-risk group would represent a higher proportion. As persons age, the high-risk group falls more often prey to mortality, and only the low-risk group is left. At that stage, no effect of preferential status on the risk of death would be measured. Such a mechanism could also explain why the effect of preferential status on use of residential care is much smaller than the association with home care. Persons enter residential care generally at age 85 or older, while first use of home care is registered for many older persons below that age. In other words, the reason that we find that persons with preferential status are not more likely to move into care homes than those without that status (and are also not more likely to get dementia) is that the former tend to die before they attain the age at which those events commonly occur. This would be an instance of what in survival analysis terminology is called ‘informative censoring’ [19]: conditional on observed variables, those persons whose observation periods are censored by dying would have been more likely to experience the event of interest (entering residential care) if they had continued to live, compared to those who do not die. It is important to stress that such an interpretation, if correct, does not change the evaluation of health inequalities in a life-course perspective. If differences in the likelihood of starting to experience health problems by socio-economic status are larger at younger than at older ages, this does not change anything for a birth cohort that will pass through all those ages.

Alternatively, one might interpret the effect of preferential status on the use of home care in terms of prices. For persons enjoying preferential status, co-payments for this kind of care are reduced, and this might induce them to use it more frequently, or at lower levels of need. The difference in prices is not negligable, about 4 € per day for standard packages of home care [24]. On the other hand, many persons receiving home care do not have to pay co-payments, irrespective of preferential status, as the nurses do not always charge these, or because those persons are covered by the system of maximum billing (which puts a ceiling on the total amount of co-payments during a calendar year). There are no co-payments for care in residential settings, so in this respect the limited effect of preferential status on residential care use is in agreement with the economic interpretation in terms of prices. In addition, there might be an income effect, as persons have to pay from their own resources the substantial costs for bed and board in care homes. Older people with low incomes might be less inclined to enter residential care for this reason, especially if they are unwilling to relinquish their own home at the same time. However, such an interpretation requires an additional explanation for why this supposed price effect would be much smaller, or non-existent, for the very old than for the not so old. Also, differential prices cannot explain why persons enjoying preferential status die at younger ages than older persons without that status. So the principle of scientific parsimony would favor the health interpretation of the effect of preferential status.

Moreover, these rival explanations have a number of different implications which can be tested. For instance, if lower co-payments would induce persons with preferential status to use home care at lower levels of need, compared to other persons, then persons with preferential status should be more likely to use home care at a low level of intensity than others, since the provider decides on the level of home care provided (subject to periodical checks by the insurer). In a logistic regression with the level of home care as the dependent variable, conditional on receiving home care, preferential status had no significant effect, however. Also, if the interpretation in terms of prices of the effect of preferential status would be correct, then within the group of persons receiving home care at a low level those having preferential status would be less likely than those without that status to make the transition to either home care at a high level, or to death. Again, in analyses of these transitions, preferential status had no significant effect (results available on request). One must keep in mind, though, that due to the much smaller sample sizes the power of the significance tests was lower than for the analyses reported in the body of the paper. Of course it is also true that these interpretations are not mutually exclusive, and both may operate in the real world.

We have also seen that the effect of preferential status is consistently smaller for women than for men. A possible reason for this finding is that preferential status is a better indicator of socio-economic status for men than for women. Almost all men in this age group have worked for most of their active lives, so a low income in old age is an indication of low earnings during that period, and therefore of less favorable occupations and educational levels. On the other hand, many women may have been housewives for a large part of their former lives, irrespective of their own education and occupation, or those of their husband. A low income in old age may be less correlated for this reason with those other indicators of socio-economic status.

## Conclusions

The results of this study generally confirm the small but growing literature on socio-economic inequality in morbidity among older persons which suggests that social inequalities in health persist into old age. We find a strong association between preferential status, our indicator of socio-economic status, and the likelihood of home care use. For residential care the association is weak for men and non-existent for women. We also find that preferential status is significantly related to the chance of getting two out five chronic conditions – COPD and diabetes, but not dementia, hip fracture and Parkinson’s disease – and with the probability of dying (not for women). For home care use and death, the association with preferential status declines with age from age 65 onwards, such that it is near zero for those aged around 90 and older.

We have argued that the most plausible explanation of these associations is in terms of health: persons with low socio-economic status and low income have worse health than those with better socio-economic status and higher income, leading to a greater likelihood of disabilities, which in turn leads to higher demand for and use of formal long-term care, both at home and in residential settings. As persons having preferential status have to pay less for formal home care, an alternative (though partial) explanation is in terms of price and income effects. Better data on the incomes of older persons, as well as on other measures of socio-economic status, e.g. by linking the data used here to social security or tax data, would make it possible to perform more formal tests of these rival interpretations. Of course, the observed associations may represent the cumulative effects of both mechanisms. Regarding the finding that the associations decline with age, we have proposed selective survival as a possible explanation. More data on morbidities, in particular on heart problems, e.g. by linking administrative data to data from the Health Interview Surveys, would help to confirm or disprove this hypothesis.

Projections of the future use of long-term care indicate that long-term care systems in Europe will face considerable challenges in meeting strongly increasing demand [25]. The results of this study suggest that reducing social inequalities in health could be one way of limiting this challenge.

## Endnotes

^a.^On 01/02/2012 the income threshold was €16306,33 (gross taxable income per year) for a single person. This amount is increased with € 3018,74 for each dependent person (i.e. each person that has to live from the same income). These amounts are regularly updated in line with increasing prices and incomes [26].

## Abbreviations

COPD: Chronic obstructive pulmonary disease; EPS: Echantillon permanent(e) steekproef (permanent sample).

## Competing interests

The authors declare that they have no competing interests.

## Authors’ contributions

KVdB carried out the analyses and drafted the manuscript. JG contributed to the study design, revised the manuscript and made a number of substantive suggestions. PW worked out the study design and revised the manuscript. All authors read and approved the final manuscript.

## Supplementary Material

Additional file 1 Cox_regressies. Click here for file

Additional file 2 Models_chronic_dead. Click here for file

Additional file 3 Models_homecare. Click here for file

Additional file 4 Models_residcare. Click here for file
